# Effectiveness of SNAPPS for improving clinical reasoning in postgraduates: randomized controlled trial

**DOI:** 10.1186/s12909-019-1670-3

**Published:** 2019-06-21

**Authors:** Vishakha Jain, Siddharth Rao, Mariya Jinadani

**Affiliations:** 1 Department of Medicine, Mahatma Gandhi Institute of Medical sciences, Sewagram, Wardha, Maharashtra 442102 India; 2 Department of Surgery, Mahatma Gandhi Institute of Medical sciences, Sewagram, Wardha, Maharashtra 442102 India; 3 Department of Physiotherapy, Seth GS medical college and KEM hospital, Mumbai, India

**Keywords:** Inpatient learning, Teaching, Postgraduates, SNAPPS, Traditional method

## Abstract

**Background:**

In-patient postgraduate teaching suffers with issues like long and unstructured presentations inclusive of a lot of historical information and time constraints due to increasing workload. A six-step pneumonic SNAPPS a learner-centered model modifies the learning encounter by condensing the reporting of facts while encouraging clinical reasoning. This study was planned with the aim to evaluate the effectiveness of SNAPPS as compared to traditional case presentation for facilitating clinical reasoning in inpatient setting. We also wanted to understand perceptions of postgraduates and teachers about this new method of case presentation.

**Methods:**

This open labeled randomized controlled trial was carried amongst the 18 residents of department of Medicine, MGIMS. The teachers and residents in the SNAPPS were sensitized to SNAPPS technique by using videos, role plays and handouts over 2 sessions of 30 min each. Twenty-seven case presentations (3/resident) were carried out in each group (total 54 case presentations). Data was recorded into validated data recording sheet after each presentation and feedback was taken from the teacher as well as residents regarding their perception.

**Results:**

The SNAPPS model heralds a change in the preceptor training, pairing faulty development and learner development as companions in education. Guided by the SNAPPS technique, students summarized patient findings concisely (7 vs. 2.7 vs. 5.22vs. 2.33, *p* = 0.0057) while maintaining the same degree of thoroughness as in traditional case presentations. The students in the SNAPPS group were clearer about their diagnostic hypothesis and compared and contrasted their different diagnosis well (2.56 vs. 1.74, *p* value =0.002). The students in the SNAPPS group initiated patient management discussion almost 20% more times as compared to the control group.

**Conclusion:**

We conclude that SNAPPS a learner centered technique for case presentations facilitated the expression of clinical diagnostic reasoning and case based uncertainties in the inpatient setting without extending the unusual length of the student case presentations. It also paved way for enhanced self-directed learning.

## Background

Medical teachers face a number of challenges in developing the clinical reasoning in postgraduate residents. Helping to develop a resident’s clinical acumen is a very complex process and is laced with challenges like complex patients, documentation requirements, increasing workloads, time constraints and productivity goals competing with the teaching time. Two frameworks for developing clinical reasoning acumen – one-minute preceptor (OMP) and SNAPPS (**S**ummarize history and findings; **N**arrow differentials; **A**nalyze differentials; **P**robe preceptor about uncertainties; **P**lan management; **S**elect case-related issues for self-study), have been well studied in the outpatient settings. These models can provide opportunities for hospitalist educators to better assess trainees, integrate regular feedback, and encourage self-directed learning. These teaching frameworks can also allow preceptors to provide more focused education to trainees without taking additional valuable time [1, 2].

The SNAPPS [3] model heralds a change in the preceptor training, pairing faulty development and learner development as companion in education. A six-step pneumonic SNAPPS, a learner-centered model, modifies the learning encounter by condensing the reporting of facts while encouraging clinical reasoning. It was developed based on cognitive learning (Bordage) and reflective practice theory (Osterman and Kottkamp) [3].

Developing clinical reasoning in a resident is a very complex process. The literature elucidates strategies to help develop this clinical reasoning and also explains the process of clinical reasoning of a novice Vis a Vis expert resident. All clinicians know that clinical reasoning of an experienced person is a well- balanced approach between pattern recognition and hypothetic-deductive approach and while that of a novice goes through hypothetic-deductive approach. Both the approaches have their own importance, but hypothetic-deductive approach more so especially in cases of undifferentiated illness**.** SNAPPS is a technique for case presentation which enables the process of hypothetic-deductive approach and also of self-directed learning. The ability to develop differential diagnosis, reason the attributes of a differential; is a process to further augment clinical reasoning. SNAPPS gives explicit steps to the postgraduate’s and hones their clinical reasoning skill and hence after thorough review of literature this technique was considered for our study.

The steps in SNAPPS technique are drawn from the cognitive rating scale developed by Connell [4]. SNAPPS has been used widely for the outpatient or office setting teaching. A small study done in pediatrics outpatient demonstrated the effectiveness of SNAPPS as a method of case presentation which improved clinical reasoning. The resident in the study perceived that the SNAPPS model was more structured, stimulating and relevant to teaching in out-patient department (OPD), was easy to follow and made them motivated for self-learning [5]. There has been only a handful cursory research utilizing SNAPPS for inpatient learning which provides a wonderful teaching learning opportunity [2]. But the long, unstructured postgraduate presentation inclusive of a lot of historical information becomes quite time consuming. The increase time of presentation also leads to the teacher offering the diagnosis passively due to time constraints and increased patient workload. Also, the increased time comprises patient care both at faculty and postgraduate level. Additionally, it does not showcase the analytical skills and deductive logic of the post graduate student to arrive to a diagnosis after including or excluding an array of differential diagnosis. So, the teaching learning opportunity in in-patient settings is lost or happens in a very haphazard manner. A review and critique of different teaching models to optimize learner centered technique in busy clinical settings concluded that compared with OMP, using SNAPPS provides learners the opportunity to be more active in their learning, including questioning the preceptors and identifying topics for asynchronous learning [6, 7]. Moreover, students can drive the content of the teaching they receive based on uncertainties they express to preceptors during case presentations [8].

Hence, this study was planned with the aim to evaluate the effectiveness of SNAPPS as compared to traditional case presentation for facilitating clinical reasoning in in-patient setting. We also wanted to understand perceptions of postgraduates and teachers about this new method of case presentation.

## Methodology

### Ethical considerations

An approval was taken from the institutional review board (IRB) before starting the study *(ref no: MGIMS/IEC/MED/46/2017 dated 8th June 2017).* Informed consent was taken from the residents before inclusion in the study.

### Study design

This study was a randomized controlled and crossover trial. The study subjects were randomized into 2 groups (process of randomization is explained below) and after completion of data acquisition a crossover was done for ethical reasons. So, after cross over the group 1 of traditional case presentations were also taught the technique of case presentation using SNAPPS.

### Study setting

This study was carried out in the in-patient or ward setting of Medicine department at Mahatma Gandhi institute of medical sciences (MGIMS).

### Study duration

The study period was from April 2017 to April 2018.

### Study population

The postgraduate students/residents of department of Medicine, MGIMS were the study population. Residents are the postgraduate students pursuing MD in Medicine after completion of their MBBS and internship. Eighteen residents of Medicine [postgraduate residents in the 1st year (JR1), 2nd year (JR2) and 3rd year (JR3)] were included in the study. Preceptors were from amongst the faculty of department of Medicine who are regularly involved in teaching postgraduates. A total of 4 preceptors were included in the study.

### Sample size

Eighteen residents were included in the study**.** The units of case presentation were considered for the data analysis. Each resident underwent a total of 3 case presentations. A total of 54 case presentations were done (27 presentations in traditional case presentation group and 27 case presentations in the SNAPPS group).

### Sampling and randomization

The study population was included based on convenient sampling amongst the residents of department of Medicine. The eighteen residents who consented were then randomized by simple randomization technique using a simple random number table. After randomization, the study population was divided into 2 groups – group 1 was the SNAPPS technique group and group 2 was the control- traditional case presentation group.

### Intervention

The intervention was the implementation of SNAPPS model as a method of case presentation in in-patient settings.

### Study methods and procedures

#### Group 1 SNAPPS group

##### Teacher’s sensitization and training

All teachers/preceptors in the SNAPPS group had a session of introduction and sensitization to SNAPPS technique 2 weeks prior to start of actual case presentation by using SNAPPS technique. This introduction and sensitization regarding SNAPPS was carried out by the principal investigator. This was a 30-min program where all teachers were shown an instructional video demonstrating the SNAPPS technique and had an opportunity to ask questions and clarify their doubts. The instructional video is a validated video used by previous studies [9]. They also received a card/handout highlighting the six steps of SNAPPS technique. There was reinforcement training session lasting for 30 min, 7 days prior to actual case presentation wherein they were given an opportunity to clarify their doubts.

##### Resident’s sensitization

Residents randomized in the SNAPPS group received a more detailed introductory and sensitization program on the SNAPPS technique. The resident’s sensitization was carried out by the principal investigator along with the sensitized teachers. This was carried out 2 weeks prior to the start of the actual presentation in 2 sessions of half hour each. The first session included –discussion of conventional method of case presentations, introduction to the concept of SNAPPS technique, role-play demonstrating SNAPPS technique, instructional video demonstrating SNAPPS technique and an opportunity to ask questions. The second session was for reinforcing the use of SNAPPS technique and was carried out 1 week before the start of presentations. They also received a card/handout highlighting the six steps of SNAPPS technique. The students were requested not to discuss the study or the SNAPPS technique with their peers.

##### SNAPPS group case presentations

After randomization 9 residents in the SNAPPS group had 3 rounds of case presentations each. A total of 27 traditional case presentations were done. About 2–3 presentations were done per week. We arrived at the number of 3 case presentations per resident after extensive deliberations with senior and experts in the field of medical education. This was decided keeping in mind – the time period of the study, number of cases to actually see the change in pattern of presentation and any change in outcome, feasibility in terms of time and other resources. The teachers trained in taking case presentation with SNAPPS method took these case presentations and recorded the data on the data-recording sheet after each presentation. The cases were routine cases admitted to the medicine department.

##### Group 2-control-conventional/traditional case presentation

There was no specific training given for conventional/traditional case presentations. The traditional case presentations are the usual presentations actually occurring during the routine teaching in inpatient setting. Each resident had 3 round of case presentation. A total of 27 traditional case presentations were done. About 2–3 presentations were done per week. The preceptor recorded the data on the data-recording sheet after each presentation. The cases were routine cases admitted to the medicine department.

##### Case selection

The category of cases were identified as per the “must know” area of level of postgraduate residents in the 1st year (JR1), 2nd year (JR2) and 3rd year (JR3). The different cases were: Anemia (general); generalized lymphadenopathy (general); Anasarca (general); fever with rash (general); jaundice with hepatosplenomegaly (abdomen); cirrhosis of liver with portal hypertension (abdomen); pleural effusion (respiratory system- RS); hydropneumothorax (RS); pleural fibrocavitory lesion (RS); rheumatic heart disease (cardiovascular system-CVS); congenital heart disease (CVS); quadriplegia (central nervous system – CNS); paraplegia(CNS), hemiplegia (CNS),monoplegia (CNS) etc. Since the study population was a mix of JR-1, JR2 and JR-3 it was necessary to give the cases as per the knowledge level of the residents. After the consensus from experts it was decided to give general and abdomen cases to JR-1; abdomen and RS cases to JR-2 and CVS and CNS cases to JR-3.

Attributes for the case required to be identified were discussed by the preceptors and included. The 9 basic clinical attributes identified from the literature which were to be included in the presentation by the students for summary conciseness and completeness are as follows: 1. demographic details; 2. chief complaints; 3. history of presenting illness; 4. relevant review of systems 5. relevant past history; 6. relevant personal history; 7. relevant physical examination; 8. relevant lab investigations; 9. relevant imaging findings.

##### Cross over

After the case presentations and data collection of both the groups was completed, a crossover was done for ethical reasons and the residents who are in the traditional case presentation group were sensitized to the SNAPPS method of case presentation using the same protocol used for sensitization of the SNAPPS group. We did not acquire any data after the cross-over.

##### Effectiveness of facilitation of clinical reasoning

The diagnostic thinking inventory (DTI) is a validated self report inventory that helps measure clinical reasoning [10]. The DTI measures two cognitive constructs that emerged from the clinical reasoning research namely flexibility in thinking and evidence for structure in memory. Flexibility in thinking refers to the use of a variety of thinking styles that can be applied during the diagnostic process. Structure in memory refers to the availability of knowledge, stored in memory, during the diagnostic process. It is assumed that availability is a direct consequence of adequate knowledge organization. The students in both the groups were asked to fill up the DTI after the last case presentation. The DTI scores of both the groups were then computed in both the categories –flexibility in thinking and structure of memory. These scores were then compared with the available scores for peers in the same group.

##### Perception of students and preceptors

The perception of students and preceptors was assessed by taking their feedback on a 5 point Likert scale ranging from strongly disagree, disagree, neutral, agree and strongly agree.

##### Assessment of outcomes

At the end of each case presentation session, outcomes were measured by assessing various dependent variables [11]. The effectiveness was studied using the outcomes (dependent variables) which included(1) summarize patient findings (total, summary, and discussion presentation times, conciseness, summary thoroughness – basic attributes and completeness); (2) providing differential diagnosis (number of diagnoses in the differential, number of basic attributes supporting the differential); (3) analysis possibilities of differential diagnosis (number of justified diagnoses and comparing-contrasting); (4) expressing uncertainties and obtaining clarification (number of student-initiated questions or discussions about uncertainties, diagnosis); (5) discussing patient management (number of student-initiated questions or discussions about management); (6) identifying case related reading (student initiated reading selections) [12]. The components included in the feedback form were – which method of case presentation did the students feel is better and why; were they able to speak out all the difficulties faced while case discussion, narration of patient management plan, were they able to identify sufficient case based learning issues for self-study, time management during case presentations etc. The components of the feedback form were decided upon after discussion and deliberations with seniors and experts in the field of medical education. These components are adapted from the very basic premise of the SNAPPS technique.

##### Statistical analysis

All the data from recording sheet and feedback forms was entered electronically using Microsoft excel. Data was analyzed using Stata software (Version 11, Stata Corporation, Texas, USA). As our sample size was less than 30 and was normally distributed; we compared means with 2-tailed unpaired *t* tests, medians with Mann Whitney *U* test and proportions with the chi square test. *P* < 0.05 was considered significant.

## Results

The teachers/preceptors recorded the performance of students during each case encounters in both the groups. Table 1 highlights the results of each dependant variable according to the outcome categories for all case encounters. Case presentations were used as the unit of analysis.

**Table 1:** Results of each dependant variable according to outcome categories in the case encounters

Outcomes	Dependant variables	SNAPPS group	Control group	*P* value
Summarize patient findings	Total presentation length (in minutes) (Mean ± SD)	7.19 ± 1.08	5.56 ± 1.12	*t* = 5.45 *p* = < 0.01
	Summary conciseness as a proportion of whole	2.28	1.60	0.6984
	Duration of summary (in minutes) (Mean ± SD)	3.15 ± 0.98	3.48 ± 0.80	*t* = − 1.36 *p* = 0.177
	Duration of discussion (in minutes) (Mean ± SD)	4.04 ± 1.25	2.07 ± 0.73	*t *= 7.05 *p* = < 0.01
Narrowing a differential diagnosis/	Summary thoroughness – number of basic clinical attributes covered (Mean ± SD)	7 ± 2.27	5.22 ± 2.33	*t *= 2.84 *p* = 0.006
	Number of diagnoses kept(Dx) in differential diagnosis (DDx) (Mean ± SD)	2.56 ± 0.75	1.74 ± 1.02	*t* = 3.33 *p* = 0.002
Analyzing differential diagnosis	Number of basic attributes in support of Dx in the DDx(Mean ± SD)	2.04 ± 1.06	1.07 ± 0.73	*t* = 3.89 *p* = < 0.01
	Number of justified Dx in the DDx (Median-IQR)	Median – 2.0 SD – 0.97 IQR (2–3)	Median – 0.00 SD – 0.82 IQR (0–1)	*p *= < 0.01*
	Number of distinct comparisons made between two diseases (Median-IQR)	Median – 2.0 SD – 1.02 IQR (2–3)	Median – 0.00 SD – 0.89 IQR (0–2)	*p* = < 0.01*
Probe preceptor- Expressing uncertainties	Number of uncertainties expressed and obtained clarifications (Mean ± SD)	2.19 ± 0.68	1.07 ± 1.04	*t* = 4.65 *p* = < 0.01
	Percentage of students seeking clarification and information by asking questions by acknowledging their uncertainties	26 (96.29%)	16 (59.26%)	*X*^2^ = 8.72 *p* = 0.0031
Discussed patient management plan	Percentage of presentations of students initiating patient management plan	27 (100%)	21 (77.8%)	*X*^2^ = 8.33 *p* = 0.004
Discussed case related topics and resources	Percentage of presentation by students initiating discussion by identifying topics and issues related to case and patient care for self directed learning	27 (100%)	9 (33.3%)	*X*^2^ = 3.0 *p* = 0.083

* Mann-Whitney U test

### Summarize the patient findings

Presentation length -The student in the SNAPPS group took on average 1.6 min more to make their entire case presentations (7.19 vs. 5.56, *p* value < **0.01**). The time taken to summarize was less in the SNAPPS group (3.15 vs. 3.48, *p* value =0.177). The time take for discussion was more in SNAPPS group as compared to control group (4.04 vs. 2.07, *p* value < **0.01).**

Summary conciseness - Students using the SNAPPS technique were more concise in their summaries (proportion of total presentation time) than students in the control group (2.28 compared to 1.6, *p* value = 0.6984).

### Narrowing differential diagnosis

Summary thoroughness (number of basic clinical attributes covered out of the 9 basic clinical attributes)- The students in the SNAPPS group reported an average of 7 clinical attributes as compared to the control group which reported on an average 5.2 clinical attributes (*p* value = **0.006**).

Number of diagnoses kept(Dx) in differential diagnosis (DDx) – students using the SNAPPS techniques expressed 1.5 times more differential diagnosis as compared to the control group (2.56 vs. 1.74, *p* value =**0.002**).

### Analyzing differential diagnosis

Number of basic attributes in support of Dx in the DDx – the students in the SNAPPS group on an average discussed approx twice more basic attributes in support of the diagnosis in their differential diagnosis as compared to the students in the control group (2.04 vs. 1.07, *p* value < **0.01**).

Number of justified Dx in the DDx – Students in the SNAPPS group justified their diagnostic possibilities twice more than the control group (median 2 vs.0, *p* value < **0.01**) by providing supporting evidence from the case summary, literature, or their previous experience.

Number of distinct comparisons made between two diseases – the students in the SNAPPS group were twice likely to make the distinct comparisons between the diseases in their diagnosis as compared to the control group (median 2 vs.0, *p* value < **0.01**).

### Probe preceptor- expressing uncertainties

The students in the SNAPPS group on an average expressed uncertainties and sought clarifications on twice more issues than as compared to the control group (2.19 vs. 1.07, *p* value < **0.01**). Almost all students in the SNAPPS group formulated uncertainties and sought clarification as compared to only 60% in the control group (96.29% vs.59.26%, *p* value =**0.0031**).

### Discussed patient management plan

Students using the SNAPPS technique initiated management discussions nearly 20% more often than students in the control group (100% versus 77.8%, *p* value =0.004).

### Identifying case related topics and resources for self study

Student-initiated selection of readings occurred among all students using the SNAPPS technique as compared to only 33% in the control group (100% versus 33.33%, *p* value =**0.083**).

Table 2 and Table 3 highlights the feedback taken from teachers and students respectively on five-point Likert scale. According to the perception of teachers, the students in the SNAPPS group fared better as compared to students in the control group as they were more systemic in examination and included more relevant examination (*p* value < 0.01); were more organized in formulating and defending differential diagnosis (*p* value < 0.01); elaborating a patient management plan (*p* value < 0.01) and managing time and identifying difficulties in presentation (*p* value < 0.01). The students in both groups had similar perception on the different variables of feedback except that the student in the SNAPPS group felt they actively initiated and identifying case related issues for self study as compared to students in control group (*p* value < 0.01).

**Table 2:** Teacher feedback (Median (IQR)

Feedback parameters	SNAPPS group	Control group	*P* value*
Concisely covered all aspects of history taking	4 (4–5)	4 (2–5)	0.039
Performed all the steps of general examination	4 (4–5)	4 (2–4)	< 0.01
Systemic examination findings were relevant and in accordance with history	4 (4–5)	4 (3–4)	< 0.01
Sequencing and formulation of differential diagnosis were well organized	4 (2–4)	2 (1–4)	< 0.01
Hypothesis of differential diagnosis matching with history and examination	4 (2–4)	1 (1–2)	< 0.01
Able to speak out all the difficulties faced while case discussion	4 (4–5)	2 (2–4)	< 0.01
Narration of patient management plan – realistic and appropriate to differential diagnosis	5 (4–5)	4 (3–4)	< 0.01
Identified sufficient case based learning issues for self study	4 (4–5)	2 (1–4)	< 0.01
Time management during case presentations	5 (4–5)	3 (2–4)	< 0.01
Uniformity and skills of presentation	4 (4–4)	4 (3–4)	0.138
Overall rating of case presentation (Mean ± SD)	6.7 ± 1.46	5.3 ± 1.75	*t* = 3.20** *p* = 0.002

* Mann-Whitney U test

** Student t test

**Table 3:** Student feedback (Median (IQR)

Feedback parameters	SNAPPS group	Control group	*P* value*
Concisely covered all aspects of history taking	4 (4–4)	4 (2–4)	0.013
Performed all the steps of general examination	4 (4–4)	4 (3–4)	0.096
Systemic examination findings were relevant and in accordance with history	4 (3–4)	4 (4–4)	0.376
Sequencing and formulation of differential diagnosis were well organized	3 (2–3)	2 (2–3)	0.624
Hypothesis of differential diagnosis matching with history and examination	3 (2–4)	2 (2–4)	0.893
Able to speak out all the difficulties faced while case discussion	4 (4–4)	4 (3–4)	0.140
Narration of patient management plan – realistic and appropriate to differential diagnosis	3 (2–4)	3 (3–4)	0.601
Identified sufficient case based learning issues for self study	4 (4–4)	3 (2–4)	< 0.01
Time management during case presentations	3 (2–3)	3 (3–4)	0.057
Uniformity and skills of presentation	3 (3–3)	3 (3–4)	0.072
Overall rating of case presentation (Mean ± SD)	5.52 ± 1.58	5.48 ± 1.12	*t* = 0.099** *p* = 0.921

* Mann-Whitney U test

** Student t test

The mean DTI scores in both the groups were comparable for both the subscales of flexibility in thinking and structure on memory. When compared to the mean score for the respondents peer group, the scores in both groups were low (Table 4).

**Table 4:** Comparison of diagnostic thinking inventory (DTI) scores

	SNAPPS group	Control group	*P* value	Mean score for the respondent’s peer group
Flexibility in thinking (max score = 126)	64.75 ± 5.03	62.79 ± 4.76	0.206	91.6
Structure of memory (max score = 120)	69.67 ± 2.39	65.23 ± 3.45	0.324	88.5

## Discussion

This study highlighted the successful introduction of the SNAPPS technique for case presentations in the busy in-patient setting. This randomized trial showed that SNAPPS a learner centered technique for case presentations facilitated the expression of clinical diagnostic reasoning and case based uncertainties in the in-patient setting without extending the unusual length of the student case presentations. Each of the six study outcomes has important implications for teaching and learning in the inpatient setting. We discuss each outcome in turn, followed by a general discussion of the implications of the results from this study.

### Summarizing patient findings and narrowing differential diagnosis

During the case encounters the students in the SNAPPS group took a little longer time for case presentations as that of the control group, but nevertheless their discussions were significantly longer and their summaries shorter. In the various case encounters the students in the SNAPPS group contained more basic clinical attributes and more differential diagnosis as compared to the control group. Guided by the SNAPPS technique, students summarized patient findings concisely while maintaining the same degree of thoroughness as in traditional case presentations.

### Analyzing differential diagnosis

In the case encounters the students in the SNAPPS group analyzed the differential diagnosis in a much better way as compared to the counterparts in the control group in the form that they had significantly more number of basic attributes to each differential diagnosis, they justified their diagnosis better and distinctly, were able to compare two diseases apart. The students in the SNAPPS group were clearer about their diagnostic hypothesis and compared and contrasted their different diagnosis well. The students in the control or conventional group lacked in their diagnostic hypothesis and were more eager to jump to management issues. This reduced communication, made it difficult for the teacher to understand the clinical reasoning of the student and also caused difficulty in giving effective feedback [11]. Clinical teachers cannot diagnose the learner’s level of diagnostic reasoning without knowing the student’s diagnostic hypotheses [11].

### Probe preceptor- expressing uncertainties

The students in the SNAPPS group expressed their uncertainties and sought clarifications from the preceptor almost twice. All students in the SNAPPS group had formulated their uncertainties almost every time. Expressing uncertainties occurred quite less in the control group. Probing of preceptor led to better discourse and immediate feedback from the teacher. Connell and colleagues [4] found that when preceptors sought their students’ thought processes during case presentations, the learners also increased their own expression of their clinical thinking. The SNAPPS technique gives the teacher insight into the clinical reasoning of students; makes space for the immediate feedback and the teaching moments depending on the students need; correct errors and reinforce good thinking [13].

### Discussed patient management plan

The students in the SNAPPS group initiated patient management discussion almost 20% more times as compared to the control group. Initiating patient management issues helps the teacher understand the level of the learner and shape subsequent management plan [10]. Yet again there is healthy space for immediate feedback; clarifying doubts and giving immediate feedback.

### Identifying case related topics and resources for self study

The identification of case related topics and resources for self study happened almost all the time only amongst the students of the SNAPPS group as compared to the control or conventional group. It has been a well noted fact that reading related to one’s own patient has a double impact in the form of fostering cognitive as well as experiential encoding in ones memories [13]. The preceptors should encourage reading habits and self directed learning. Self directed reflective learning also enhances and helps the student build upon their existing knowledge; understanding the lacunae in their knowledge and actively seeking resources and help to fill up these lacunae; which in long term definitely leads to better knowledge and better and improved patient care.

### Effectiveness of clinical reasoning

We used DTI after the 3rd and final case presentations by students in both the groups. The scores were similar in both the groups, were comparable to each other and were low as compared to the mean score of the respondents in the peer group. DTI is an excellent tool for assessing case-specific reasoning and feedback, it is not meant to be used as a “generic” measure of diagnostic reasoning because diagnostic reasoning is highly case specific. Inter-case correlation coefficients of problem-solving abilities are typically in the 0.1 to 0.3 range. The DTI, in accordance with case specificity, is best used as a debriefing instrument in relation to one or two specific cases, not as a generic measure [14].

## Conclusions

The SNAPPS technique for case presentation in the inpatient setting enhances expression of clinical reasoning without having much effect on the time for case presentations. It needs brief faculty development for being able to facilitate this technique and an extensive student development for being able to use this technique. But this technique provides explicit steps to the students and the responsibility of expressing their clinical reasoning, expressing uncertainties, probing the preceptors and identifying issues for self study falls on the shoulders of students as a default just to be facilitated by trained mentors.

## Data Availability

The datasets used and/or analyzed during the current study are available from the corresponding author on request.

## Abbreviations

CNS: Central nervous system
CVS: Cardiovascular system
DDx: Differential diagnosis
DTI: Diagnostic thinking inventory
Dx: Number of diagnoses kept
IRB: Institutional review board
JR1, JR2, JR3: Postgraduate residents in the 1st year (JR1), 2nd year (JR2) and 3rd year (JR3)
MGIMS: Mahatma Gandhi institute of medical sciences
OMP: One-minute preceptor
OPD: Outpatient department
RS: Respiratory system
SNAPPS: **S**ummarize history and findings; **N**arrow differentials; **A**nalyze differentials; **P**robe preceptor about uncertainties; **P**lan management; **S**elect case-related issues for self-study
